# Supplementary material for: Standardized mean differences cause funnel plot distortion in publication bias assessments
Source: eLife. 2017 Sep 8;6:e24260. doi: 10.7554/eLife.24260 (PMC5621838; doi:10.7554/eLife.24260)
Supplement: Supplementary file 1. [file elife-24260-supp1.docx]

| **Supplemental table 1: Cohen’s d and Hedges’ g, as well as Egger’s test and Begg and Mazumdar’s test, perform similar in multiple illustrative scenario’s (simulation 1)** | | | | | | | | |
| --- | --- | --- | --- | --- | --- | --- | --- | --- |
| **Total study n** | **Δμ** | **No. of studies in MA** | **Effect measure** | **% of simulations with Egger’s p<0.05** | **% of simulations with Begg and Mazumdar’s p<0.05** | **Mean no. of studies filled by T&F (min-max)** | **Mean original overall effect size [95%CI]** | **Mean overall effect size after T&F [95%CI]** |
| 12-30 | 0 | 30 | RMD | 6.2% | 5.6% | 2.1 (0-11) | 0.74[-12.2, 11.3] | 0.0[-3.8, 3.6] |
|  |  |  | SMD(*d*) | 9.3% | 7.9% | 1.7 (0-10) | 0.1[-1.2, 1.5] | 0.0[-0.34, 0.33] |
|  |  |  | SMD(*g*) | 9.3% | 7.7% | 1.6 (0-10) | 0.1[-1.1, 1.4] | 0.0[-0.36, 0.33] |
| 12-30 | 5 | 30 | RMD | 4.9% | 4.8% | 2.1 (0-10) | 5.3[-3.4, 19.1) | 5.0 [1.2, 9.6] |
|  |  |  | SMD(*d*) | 20.2% | 18.3% | 2.6 (0-10) | 0.58[-0.3 – 2.3] | 0.44 [0.11, 0.74] |
|  |  |  | SMD(*g*) | 19.5% | 17.3% | 2.4 (0-10) | 0.55[-0.4 – 2.2] | 0.43 [0.11 – 0.74] |
| 12-30 | 10 | 30 | RMD | 4.6% | 4.0% | 2.0 (0-10) | 11.2[1.2, 20.4] | 10.0[5.4, 13.5] |
|  |  |  | SMD(*d*) | 67.5% | 58.0% | 4.8 (0-10) | 1.21[0.2, 2.4] | 0.88[0.5, 1.2] |
|  |  |  | SMD(*g*) | 67.2% | 56.9% | 4.4 (0-10) | 1.16[0.2, 2.4] | 0.85[0.5, 1.2] |
| 12-30 | 0 | 300 | RMD | 4.8% | 5.0% | 25.4 (0-62) | 0.0[-15.2, 12.3] | 0.0[-2.1, 2.3] |
|  |  |  | SMD(*d*) | 9.5% | 7.6% | 17.4 (0-55) | 0.0[-2.0, 1.7] | 0.0[-0.2, 0.2] |
|  |  |  | SMD(*g*) | 9.8% | 7.8% | 18.8 (0-57) | 0.0[-1.9, 1.6] | 0.0[-0.2, 0.2] |
| 12-30 | 5 | 300 | RMD | 5.5% | 5.1% | 25.1 (0-65) | 5.5[-10.2, 23.7] | 5.0 [3.0, 6.8] |
|  |  |  | SMD(*d*) | 96.8% | 96.0% | 47.7 (0-72) | 0.57[-1.1, 2.5] | 0.38 [0.29, 0.52] |
|  |  |  | SMD(*g*) | 96.0% | 94.9% | 47.3 (0-70) | 0.55[-1.1, 2.3] | 0.37 [0.28, 0.50] |
| 12-30 | 10 | 300 | RMD | 5.9% | 5.5% | 25.8 (0-61) | 10.3[-11.1, 29.0] | 10.0[7.9, 12.3] |
|  |  |  | SMD(*d*) | 100% | 100% | 63.1 (43-77) | 1.1[-1.4, 3.2] | 0.83[0.73, 0.94] |
|  |  |  | SMD(*g*) | 100% | 100% | 61.5 (40-76) | 1.0[-1.4, 3.1] | 0.80[0.70, 0.89] |
| 12-30 | 0 | 3000 | RMD | 5.4% | 5.8 | 249 (0-453) | 0.0[-18.6, 17.9] | 0.0[-1.4, 1.3] |
|  |  |  | SMD(*d*) | 8.4% | 7.4% | 153.8 (0-355) | 0.0[-1.9, 2.4] | 0.0[-0.1, 0.1] |
|  |  |  | SMD(*g*) | 8.7% | 7.6% | 175.1 (0-386) | 0.0[-2.1, 2.6] | 0.0[-0.1, 0.1] |
| 12-30 | 5 | 3000 | RMD | 4.4% | 3.9% | 252 (0-475) | 4.9 (-13.0, 21.1) | 5.0 (3.7, 6.4) |
|  |  |  | SMD(*d*) | 100% | 100% | 492 (417-572) | 0.51 [-1.8, 3.1] | 0.37 [0.34, 0.41] |
|  |  |  | SMD(*g*) | 100% | 100% | 492 (417-565) | 0.49 [-1.7, 2.9] | 0.36 [0.33, 0.39] |
| 12-30 | 10 | 3000 | RMD | 5.0% | 4.6% | 250 (0-456) | 10.0[-7, 27] | 10.0[8.6, 11.3] |
|  |  |  | SMD(*d*) | 100% | 100% | 635 (584 – 688) | 1.0[-0.7, 4.8] | 0.83[0.8, 0.9] |
|  |  |  | SMD(*g*) | 100% | 100% | 620 (568 – 669) | 1.0[-0.7, 4.5] | 0.79[0.8, 0.8] |
| 60-320 | 0 | 30 | RMD | 4.7% | 5.0% | 2.4 (0-10) | -0.2[-3.8, 3.3] | 0.0[-1.3, 1.3] |
|  |  |  | SMD(*d*) | 5.0% | 5.3% | 2.4 (0-10) | 0.0[-0.4, 0.4] | 0.0[-0.1, 0.1] |
|  |  |  | SMD(*g*) | 5.0% | 5.3% | 2.4 (0-10) | 0.0[-0.4, 0.4] | 0.0[-0.1, 0.1] |
| 60-320 | 5 | 30 | RMD | 3.8% | 4.3% | 2.2 (0-10) | 4.8 (1.9-7.6) | 5.0 (3.8 – 6.1) |
|  |  |  | SMD(*d*) | 5.6% | 4.9% | 2.4 (0-13) | 0.48 (0.2 – 0.8) | 0.5 (0.4 – 0.6) |
|  |  |  | SMD(*g*) | 5.2% | 4.9% | 2.4 (0-13) | 0.48 (0.2 – 0.8) | 0.5 (0.4 – 0.6) |
| 60-320 | 10 | 30 | RMD | 5.9% | 4.7% | 2.4 (0-10) | 10.0[6.7, 14.0] | 10.0[8.7, 11.2] |
|  |  |  | SMD(*d*) | 8.3% | 6.5% | 2.7 (0-10) | 1.0[0.6, 1.3] | 1.0[0.8, 1.1] |
|  |  |  | SMD(*g*) | 7.9% | 6.0% | 2.6 (0-10) | 1.0[0.6, 1.3] | 1.0[0.8, 1.1] |
| 60-320 | 0 | 300 | RMD | 4.4% | 4.0% | 18.9 (0-58) | 0.1[-3.7, 5.5] | 0.0[-0.5, 0.6] |
|  |  |  | SMD(*d*) | 4.6% | 4.1% | 18.0 (0-58 | 0.0[-0.4, 0.5] | 0.0[-0.1, 0.1] |
|  |  |  | SMD(*g*) | 4.6% | 4.1% | 17.3 (0-58) | 0.0[-0.4, 0.5] | 0.0[-0.1, 0.1] |
| 60-320 | 5 | 300 | RMD | 4.7% | 4.2% | 17.8 (0-63) | 4.9 [0.0, 9.7] | 5.0[4.4, 5.6] |
|  |  |  | SMD(*d*) | 14.2% | 12.1% | 21.2 (0-60) | 0.49[(0.0, 0.9] | 0.49[0.4, 0.5] |
|  |  |  | SMD(*g*) | 11.8% | 10.1% | 20.7 (0-60) | 0.49 [0.0, 0.9] | 0.49[0.4, 0.5] |
| 60-320 | 10 | 300 | RMD | 6.2% | 6.0% | 18.4 (0-63) | 10.1[4.8, 16.5] | 10.0[9.4, 10.6] |
|  |  |  | SMD(*d*) | 40.2% | 35.2% | 27.9 (0-71 | 1.0[0.5, 1.7] | 0.97[0.9, 1.0] |
|  |  |  | SMD(*g*) | 33.9% | 30.2% | 29.5 (0-71) | 1.0[0.5, 1.7] | 0.97[0.9, 1.0] |
| 60-320 | 0 | 3000 | RMD | 5.3% | 5.6% | 140.0 (0-367) | 0.0[-6.5, 5.6] | 0.0[-0.3, 0.3] |
|  |  |  | SMD(*d*) | 5.4% | 5.7% | 132.4 (0-347) | 0.0[-0.7, 0.6] | 0.0[0.0, 0.0] |
|  |  |  | SMD(*g*) | 5.4% | 5.7% | 136.6 (0-348) | 0.0[-0.7, 0.6] | 0.0[0.0, 0.0] |
| 60-320 | 5 | 3000 | RMD | 4.7% | 4.5% | 143 (0 – 331) | 5.0 [-1.4, 11.3] | 5.0 [4.7, 5.3] |
|  |  |  | SMD(*d*) | 79.5% | 72.9% | 253 (0-402) | 0.5 [-0.1, 1.2] | 0.48 [0.46, 0.51] |
|  |  |  | SMD(*g*) | 69.0% | 64.1% | 243 (0-391) | 0.5 [-0.1, 1.2] | 0.48 [0.46, 0.51] |
| 60-320 | 10 | 3000 | RMD | 5.0% | 4.7% | 135.8 (0-340) | 10.0[4.6, 16.2] | 10.0[9.7, 10.3] |
|  |  |  | SMD(*d*) | 99.8% | 99.8% | 349.3 (201-475) | 1.0[0.47, 1.62] | 0.97[0.96, 0.98] |
|  |  |  | SMD(*g*) | 99.7% | 99.4% | 334.5 (168-464) | 1.0[0.47, 1.61] | 0.97[0.95, 0.98] |
| n = sample size; Δμ = difference in normal distribution means between control and intervention group; no. = number; MA = meta-analysis; T&F = trim and fill analysis; RMD = raw mean difference; SMD = standardized mean difference; SD = standard deviation | | | | | | | | |
