# Supplementary material for: Standardized mean differences cause funnel plot distortion in publication bias assessments
Source: eLife. 2017 Sep 8;6:e24260. doi: 10.7554/eLife.24260 (PMC5621838; doi:10.7554/eLife.24260)
Supplement: Supplementary file 2. [file elife-24260-supp2.docx]

**Supplementary File 2: supplemental equations**

**Hedges’*g***

Hedges’ *g*[1] is a popular modification of Cohen’s *d*[2] that corrects for biases due to small sample sizes. Hedges’ *g* can be calculated by multiplying Cohen’s *d* with the conversion factor *J*. Thus,

$$Hedges^{'}g=J x Cohen^{'}s d$$

, where

$$J=1- \frac{3}{4 (n{}_{ctrl}+n_{int}-2)-1}$$

The standard error (SE) of Hedges’ *g* can be approximated by:

$${SE}_{Hedges^{'}g}= \sqrt{J^{2}* \frac{\left( n_{ctrl}+n_{int} \right)}{n_{ctrl}* n_{int}}+\frac{{SMD}^{2}}{2*\left( n_{ctrl}+n_{int} \right)}}$$

1. Cohen J (1988) Statistical power analysis for the behavioral sciences (2nd ed.). Hillsdale, NJ: Lawrence Erlbaum.

2. Hedges LV (1981) Distribution Theory for Glass's Estimator of Effect Size and Related Estimators. Journal of Educational Statistics 6: 107.

**Normalised mean difference (NMD)**

In many cases “normal” animals do not have a lesion, and equation 6 and 7 simplify to respectively:

$NMD=100\%\times\frac{\left( M_{int} \right)-\left( M_{ctrl} \right)}{\left( M_{ctrl} \right)}$ (6a)

and

${SE}_{NMD}=\sqrt{\frac{({100*\frac{{SD}_{ctrl}}{M_{ctrl}})}^{2}}{n_{ctrl}} + \frac{({100*\frac{{SD}_{int}}{M_{int}})}^{2}}{n_{int}}}$ (7a)
