# Supplementary material for: Standardized mean differences cause funnel plot distortion in publication bias assessments
Source: eLife. 2017 Sep 8;6:e24260. doi: 10.7554/eLife.24260 (PMC5621838; doi:10.7554/eLife.24260)
Supplement: Supplementary file 3. [file elife-24260-supp3.pdf]

Supplementary File 3: R-scripts

```
##Welcome to Peter-Paul's meta-simulation Headquarters. I hope you will enjoy
 yourself :) This script is adapted from 'http://www.r-bloggers.com/practicing-meta-
 analytic-thinking-through-simulations/' with courtesy and huge thanks to Daniel
 Lakens (TuEindhoven)!!

library(ggplot2)
library(MBESS)
library(pwr)
library(meta)
library(xlsx)
cbbPalette<-c("#E69F00", "#56B4E9", "#009E73", "#F0E442", "#0072B2", "#D55E00",
 "#CC79A7")

SIMULATION SCRIPT
mx<-40 #Set mean sample Therapy
sdx<-10 #Set standard deviations sample Therapy
my<-30 #Set mean sample Control
sdy<-10 #Set standard deviation sample Control
mz<-70 #Set mean sample sham group
sdz<-4 #Set standard deviation for Sham group

nSims2 <- 1000 #set number of simulations of these meta-analysis
nSims <- 3000 #set number of simulated studies in any meta-analysis
ES.rmd <-numeric(nSims) #set up empty container for all simulated ES (RMDs)
es.d2 <-numeric(nSims) #set up empty container for all simulated ES (hedges g)
es.d <-numeric(nSims) #set up empty container for all simulated ES (cohen's d)
es.nmd <-numeric(nSims) #set up empty container for all simulated NMDs
pvalue<-numeric(nSims) #set up empty container for all simulated p-value's

p.eggerRMDall<-numeric(nSims2)
p.eggerRMDpub<-numeric(nSims2)
p.eggerSMDall<-numeric(nSims2)
p.eggerSMDpub<-numeric(nSims2)
p.eggerSMDcohenall<-numeric(nSims2)
p.eggerRMDallfp<-numeric(nSims2)
p.eggerRMDpubfp<-numeric(nSims2)
p.eggerSMDallfp<-numeric(nSims2)
p.eggerSMDcohenallfp<-numeric(nSims2)
p.eggerSMDpubfp<-numeric(nSims2)
p.eggerNMDall<-numeric(nSims2)
p.eggerNMDpub<-numeric(nSims2)
p.eggerNMDallfp<-numeric(nSims2)
p.eggerNMDpubfp<-numeric(nSims2)
totalfillRMDall<-numeric(nSims2)
totalfillRMDpub<-numeric(nSims2)
totalfillSMDall<-numeric(nSims2)
totalfillSMDpub<-numeric(nSims2)
totalfillSMDcohenall<-numeric(nSims2)
totalfillRMDallfp<-numeric(nSims2)
totalfillRMDpubfp<-numeric(nSims2)
totalfillSMDallfp<-numeric(nSims2)
totalfillSMDcohenallfp<-numeric(nSims2)
totalfillSMDpubfp<-numeric(nSims2)
p.rankRMDall<-numeric(nSims2)
p.rankRMDpub<-numeric(nSims2)
p.rankSMDall<-numeric(nSims2)
p.rankSMDcohenall<-numeric(nSims2)
p.rankSMDpub<-numeric(nSims2)
p.rankRMDallfp<-numeric(nSims2)
p.rankRMDpubfp<-numeric(nSims2)
p.rankSMDallfp<-numeric(nSims2)
p.rankSMDcohenallfp<-numeric(nSims2)
```

```
p.rankSMDpubfp<-numeric(nSims2)
p.rankNMDall<-numeric(nSims2)
p.rankNMDpub<-numeric(nSims2)
p.rankNMDallfp<-numeric(nSims2)
p.rankNMDpubfp<-numeric(nSims2)
MARMDall<-numeric(nSims2)
MARMDpub<-numeric(nSims2)
MASMDall<-numeric(nSims2)
MASMDcohenall<-numeric(nSims2)
MASMDpub<-numeric(nSims2)
MARMDallfp<-numeric(nSims2)
MARMDpubfp<-numeric(nSims2)
MASMDallfp<-numeric(nSims2)
MASMDcohenallfp<-numeric(nSims2)
MASMDpubfp<-numeric(nSims2)
MANMDall<-numeric(nSims2)
MANMDpub<-numeric(nSims2)
MANMDallfp<-numeric(nSims2)
MANMDpubfp<-numeric(nSims2)

adjustedMASMD<-numeric(nSims2)
adjustedMASMDcohen<-numeric(nSims2)
adjustedMARMD<-numeric(nSims2)

IsqRMD<-numeric(nSims2)
TausqRMD<-numeric(nSims2)
IsqSMD<-numeric(nSims2)
TausqSMD<-numeric(nSims2)

## FOR REVISIONS ##

SSn1 <-numeric(nSims) #set up empty container for random sample sizes group 1
SSn2 <-numeric(nSims) #set up empty container for random sample sizes group 2
SSn3 <-numeric(nSims) #set up empty container for random sample sizes group Sham

for(j in 1:nSims2){ #for all sims

for(i in 1:nSims){ #for each study in the meta-analysis
  SampleSize1<-sample(7:14, 1,replace=TRUE) #randomly draw a sample between 7 and 14.
 ('clinical' = 40:150)
  SampleSize2<-sample(SampleSize1+(-2:2), 1,replace=TRUE) #randomly determine number
 of controls -2:+2 from sample control group ('clinical'=-20:+20)
  SampleSize3<-sample(4:6, 1, replace=TRUE) #random sample for sham group between 4
 and 6

x<-rnorm(n = SampleSize1, mean = mx, sd = sdx) #produce simulated participants
y<-rnorm(n = SampleSize2, mean = my, sd = sdy) #produce simulated participants
z<-rnorm(n = SampleSize3, mean = mz, sd = sdz) #produce simulated participants sham

SSn1[i]<-SampleSize1 #save sample size group 1
SSn2[i]<-SampleSize2 #save sample size group 2
SSn3[i]<-SampleSize3 #save sample size group sham

ES.rmd[i]<-mean(x)-mean(y)

es.d[i]<-smd(Mean.1= mean(x), Mean.2=mean(y), s.1=sd(x), s.2=sd(y), n.1=SampleSize1,
 n.2=SampleSize2, Unbiased=FALSE) #Use MBESS to calc d unbiased (Unbiased=TRUE Hedges
 g) and biased (biased=FALSE Cohen's d)

es.d2[i]<-smd(Mean.1= mean(x), Mean.2=mean(y), s.1=sd(x), s.2=sd(y), n.1=SampleSize1,
 n.2=SampleSize2, Unbiased=TRUE) #Use MBESS to calc d unbiased (Unbiased=TRUE Hedges
 g) and biased (biased=FALSE Cohen's d)
```

```
es.nmd[i]<-100* (   (mean(y)-mean(z)) -  (mean(x)-mean(z)) )    /    (mean(y)-
 mean(z))

TESTING<-t.test(x,y)
pvalue[i]<-TESTING$p.value
}

#Insert effect sizes and sample sizes
n1<-c(SSn1)
n2<-c(SSn2)
J<-1-3/(4*( SSn1+ SSn2-2)-1) #correction for bias = Hedges g variance (unbiased!)
es.rmd.v <- (sd(x)^2/ SSn1)+(sd(y)^2/SSn2)
es.rmd.v.miss <- (sd(x)^2/ SSn1)+(sd(y)^2/SSn2)
es.d.v <-(((SSn1+SSn2)/(SSn1*SSn2))+(es.d^2/(2*(SSn1+SSn2))))  #cohen's d
es.d.v2<-(((SSn1+SSn2)/(SSn1*SSn2))+(es.d2^2/(2*(SSn1+SSn2))))*J^2  #hedges g
es.d.v2fixed<-(((SSn1+SSn2)/(SSn1*SSn2))+(1^2/(2*(SSn1+SSn2))))*J^2 #corrected!!!
n.fp <-1/sqrt(SSn1+SSn2) #for correction in funnelplot
n.precisionnew<-(((SSn1+SSn2)/(SSn1*SSn2)))
sd.nmd.c<- 100*  ( ( sd(y)/   (mean(y)-mean(z)) ) )
sd.nmd.rx<- 100*  ( (sd(x)/   (mean(y)-mean(z)) ) )

#Calculate Standard Errors ES
d.se<-sqrt(es.d.v) #cohen's d
d.se2<-sqrt(es.d.v2)   #hedges g
d.se2fixed<-sqrt(es.d.v2fixed)   #hedges g
rmd.se<-sqrt(es.rmd.v)
nmd.se<-sqrt(((sd.nmd.c^2)/SSn2)+((sd.nmd.rx^2)/SSn1))

alldata <-data.frame(ES.rmd,rmd.se,es.d,d.se,es.d2, d.se2, d.se2fixed, es.nmd,
 nmd.se,n.fp,n.precisionnew, pvalue)

subset1<-alldata[ sample( which(alldata$pvalue<=0.05),
 round(0.9*length(which(alldata$pvalue<=0.05)))), ]
summary(alldata$pvalue<0.05)
summary(subset1)
nrow(subset1)

subset2<-alldata[ sample( which(alldata$pvalue>0.05 & alldata$pvalue<=0.10),
 round(0.5*length(which(alldata$pvalue>0.05 & alldata$pvalue<=0.10)))), ]
summary(alldata$pvalue>0.05 & alldata$pvalue<=0.10)
nrow(subset2)

subset3<-alldata[ sample( which(alldata$pvalue>0.10),
 round(0.1*length(which(alldata$pvalue>0.10)))), ]
summary(alldata$pvalue>0.10)
nrow(subset3)

pubbiasdata<-rbind(subset1,subset2,subset3)
nrow(pubbiasdata)

metaRMDall<-metagen(ES.rmd,rmd.se,data=alldata)
MARMDall<-metaRMDall$TE
eggerRMDall<-metabias(metaRMDall)
p.eggerRMDall[j]<-eggerRMDall$p.value
TFRMDall<-trimfill(metaRMDall)
totalfillRMDall[j]<-TFRMDall$k0
adjustedMARMD[j]<-TFRMDall$TE.random
rankRMDall<-metabias(metaRMDall,method.bias="rank")
p.rankRMDall[j]<-rankRMDall$p.value
IsqRMD[j]<-metaRMDall$I2
TausqRMD[j]<-metaRMDall$tau^2


metaRMDpub<-metagen(ES.rmd,rmd.se, data=pubbiasdata)
```

```
MARMDpub<-metaRMDpub$TE
eggerRMDpub<-metabias(metaRMDpub)
p.eggerRMDpub[j]<-eggerRMDpub$p.value
TFRMDpub<-trimfill(metaRMDpub)
totalfillRMDpub[j]<-TFRMDpub$k0
rankRMDpub<-metabias(metaRMDpub,method.bias="rank")
p.rankRMDpub[j]<-rankRMDpub$p.value

metaSMDall<-metagen(es.d2, d.se2, data=alldata)
MASMDall<-metaSMDall$TE
eggerSMDall<-metabias(metaSMDall)
p.eggerSMDall[j]<-eggerSMDall$p.value
TFSMDall<-trimfill(metaSMDall)
adjustedMASMD[j]<-TFSMDall$TE.random
totalfillSMDall[j]<-TFSMDall$k0
rankSMDall<-metabias(metaSMDall,method.bias="rank")
p.rankSMDall[j]<-rankSMDall$p.value
IsqSMD[j]<-metaSMDall$I2
TausqSMD[j]<-metaSMDall$tau^2

metaSMDcohenall<-metagen(es.d, d.se, data=alldata)
MASMDcohenall<-metaSMDcohenall$TE
eggerSMDcohenall<-metabias(metaSMDcohenall)
p.eggerSMDcohenall[j]<-eggerSMDcohenall$p.value
TFSMDcohenall<-trimfill(metaSMDcohenall)
adjustedMASMDcohen[j]<-TFSMDcohenall$TE.random
totalfillSMDcohenall[j]<-TFSMDcohenall$k0
rankSMDcohenall<-metabias(metaSMDcohenall,method.bias="rank")
p.rankSMDcohenall[j]<-rankSMDcohenall$p.value

metaSMDfixed<-metagen(es.d2, d.se2fixed, data=alldata)
####

metaSMDpub<-metagen(es.d2,d.se2, data=pubbiasdata)
eggerSMDpub<-metabias(metaSMDpub)
p.eggerSMDpub[j]<-eggerSMDpub$p.value
TFSMDpub<-trimfill(metaSMDpub)
totalfillSMDpub[j]<-TFSMDpub$k0
rankSMDpub<-metabias(metaSMDpub,method.bias="rank")
p.rankSMDpub[j]<-rankSMDpub$p.value

metaRMDallfp<-metagen(ES.rmd,n.fp,data=alldata)
eggerRMDallfp<-metabias(metaRMDallfp)
p.eggerRMDallfp[j]<-eggerRMDallfp$p.value
TFRMDallfp<-trimfill(metaRMDallfp)
totalfillRMDallfp[j]<-TFRMDallfp$k0
rankRMDallfp<-metabias(metaRMDallfp,method.bias="rank")
p.rankRMDallfp[j]<-rankRMDallfp$p.value

metaRMDpubfp<-metagen(ES.rmd,n.fp, data=pubbiasdata)
eggerRMDpubfp<-metabias(metaRMDpubfp)
p.eggerRMDpubfp[j]<-eggerRMDpubfp$p.value
TFRMDpubfp<-trimfill(metaRMDpubfp)
totalfillRMDpubfp[j]<-TFRMDpubfp$k0
rankRMDpubfp<-metabias(metaRMDpubfp,method.bias="rank")
p.rankRMDpubfp[j]<-rankRMDpubfp$p.value

metaSMDallfp<-metagen(es.d2, n.fp, data=alldata)
eggerSMDallfp<-metabias(metaSMDallfp)
p.eggerSMDallfp[j]<-eggerSMDallfp$p.value
TFSMDallfp<-trimfill(metaSMDallfp)
totalfillSMDallfp[j]<-TFSMDallfp$k0
rankSMDallfp<-metabias(metaSMDallfp,method.bias="rank")
p.rankSMDallfp[j]<-rankSMDallfp$p.value
```

```
metaSMDcohenallfp<-metagen(es.d, n.fp, data=alldata)
eggerSMDcohenallfp<-metabias(metaSMDcohenallfp)
p.eggerSMDcohenallfp[j]<-eggerSMDcohenallfp$p.value
TFSMDcohenallfp<-trimfill(metaSMDcohenallfp)
totalfillSMDcohenallfp[j]<-TFSMDcohenallfp$k0
rankSMDcohenallfp<-metabias(metaSMDcohenallfp,method.bias="rank")
p.rankSMDcohenallfp[j]<-rankSMDcohenallfp$p.value

metaSMDpubfp<-metagen(es.d2,n.fp, data=pubbiasdata)
eggerSMDpubfp<-metabias(metaSMDpubfp)
p.eggerSMDpubfp[j]<-eggerSMDpubfp$p.value
TFSMDpubfp<-trimfill(metaSMDpubfp)
totalfillSMDpubfp[j]<-TFSMDpubfp$k0
rankSMDpubfp<-metabias(metaSMDpubfp,method.bias="rank")
p.rankSMDpubfp[j]<-rankSMDpubfp$p.value

metaNMDall<-metagen(es.nmd,nmd.se,data=alldata)
eggerNMDall<-metabias(metaNMDall)
p.eggerNMDall[j]<-eggerNMDall$p.value
rankNMDall<-metabias(metaNMDall,method.bias="rank")
p.rankNMDall[j]<-rankNMDall$p.value

metaNMDallfp<-metagen(es.nmd,n.fp,data=alldata)
eggerNMDallfp<-metabias(metaNMDallfp)
p.eggerNMDallfp[j]<-eggerNMDallfp$p.value
rankNMDallfp<-metabias(metaNMDallfp,method.bias="rank")
p.rankNMDallfp[j]<-rankNMDallfp$p.value

metaNMDpub<-metagen(es.nmd,nmd.se,data=pubbiasdata)
eggerNMDpub<-metabias(metaNMDpub)
p.eggerNMDpub[j]<-eggerNMDpub$p.value
rankNMDpub<-metabias(metaNMDpub,method.bias="rank")
p.rankNMDpub[j]<-rankNMDpub$p.value

metaNMDpubfp<-metagen(es.nmd,n.fp,data=pubbiasdata)
eggerNMDpubfp<-metabias(metaNMDpubfp)
p.eggerNMDpubfp[j]<-eggerNMDpubfp$p.value
rankNMDpubfp<-metabias(metaNMDpubfp,method.bias="rank")
p.rankNMDpubfp[j]<-rankNMDpubfp$p.value

}

sum(p.eggerRMDall<0.05)
sum(p.eggerSMDall<0.05)
sum(p.eggerSMDcohenall<0.05)

sum(p.rankRMDall<0.05)
sum(p.rankSMDall<0.05)
sum(p.rankSMDcohenall<0.05)

mean(totalfillRMDall)
min(totalfillRMDall)
max(totalfillRMDall)

mean(totalfillSMDall)
min(totalfillSMDall)
max(totalfillSMDall)

median(IsqRMD)
min(IsqRMD)
max(IsqRMD)

median(TausqRMD)
```

```
min(TausqRMD)
max(TausqRMD)

mean(totalfillSMDcohenall)
min(totalfillSMDcohenall)
max(totalfillSMDcohenall)

mean(MARMDall)
min(MARMDall)
max(MARMDall)

mean(MASMDall)
min(MASMDall)
max(MASMDall)

median(IsqSMD)
min(IsqSMD)
max(IsqSMD)

median(TausqSMD)
min(TausqSMD)
max(TausqSMD)

mean(MASMDcohenall)
min(MASMDcohenall)
max(MASMDcohenall)

mean(adjustedMARMD)
min(adjustedMARMD)
max(adjustedMARMD)

mean(adjustedMASMDcohen)
min(adjustedMASMDcohen)
max(adjustedMASMDcohen)

mean(adjustedMASMD)
min(adjustedMASMD)
max(adjustedMASMD)

## EGGER's TEST##
sum(p.eggerRMDall<0.05)
sum(p.eggerRMDall>0.05)
median(p.eggerRMDall)
min(p.eggerRMDall)
max(p.eggerRMDall)

sum(p.eggerRMDpub>0.05)
sum(p.eggerRMDpub<0.05)
median(p.eggerRMDpub)
min(p.eggerRMDpub)
max(p.eggerRMDpub)

sum(p.eggerSMDall<0.05)
sum(p.eggerSMDall>0.05)
median(p.eggerSMDall)
min(p.eggerSMDall)
max(p.eggerSMDall)

sum(p.eggerSMDcohenall<0.05)
sum(p.eggerSMDcohenall>0.05)
median(p.eggerSMDcohenall)
min(p.eggerSMDcohenall)
max(p.eggerSMDcohenall)
```

```
sum(p.eggerSMDpub>0.05)
sum(p.eggerSMDpub<0.05)
median(p.eggerSMDpub)
min(p.eggerSMDpub)
max(p.eggerSMDpub)

sum(p.eggerRMDallfp<0.05)
sum(p.eggerRMDallfp>0.05)
median(p.eggerRMDallfp)
min(p.eggerRMDallfp)
max(p.eggerRMDallfp)

sum(p.eggerRMDpubfp>0.05)
sum(p.eggerRMDpubfp<0.05)
median(p.eggerRMDpubfp)
min(p.eggerRMDpubfp)
max(p.eggerRMDpubfp)

sum(p.eggerSMDallfp<0.05)
sum(p.eggerSMDallfp>0.05)
median(p.eggerSMDallfp)
min(p.eggerSMDallfp)
max(p.eggerSMDallfp)

sum(p.eggerSMDpubfp>0.05)
sum(p.eggerSMDpubfp<0.05)
median(p.eggerSMDpubfp)
min(p.eggerSMDpubfp)
max(p.eggerSMDpubfp)

sum(p.eggerNMDall<0.05)
sum(p.eggerNMDall>0.05)
median(p.eggerNMDall)
min(p.eggerNMDall)
max(p.eggerNMDall)

sum(p.eggerNMDpub>0.05)
sum(p.eggerNMDpub<0.05)
median(p.eggerNMDpub)
min(p.eggerNMDpub)
max(p.eggerNMDpub)

sum(p.eggerNMDallfp<0.05)
sum(p.eggerNMDallfp>0.05)
median(p.eggerNMDallfp)
min(p.eggerNMDallfp)
max(p.eggerNMDallfp)

sum(p.eggerNMDpubfp>0.05)
sum(p.eggerNMDpubfp<0.05)
median(p.eggerNMDpubfp)
min(p.eggerNMDpubfp)
max(p.eggerNMDpubfp)

## RANK TEST##
sum(p.rankRMDall<0.05)
sum(p.rankRMDall>0.05)
median(p.rankRMDall)
min(p.rankRMDall)
max(p.rankRMDall)

sum(p.rankRMDpub>0.05)
sum(p.rankRMDpub<0.05)
median(p.rankRMDpub)
```

```
min(p.rankRMDpub)
max(p.rankRMDpub)

sum(p.rankSMDall<0.05)
sum(p.rankSMDall>0.05)
median(p.rankSMDall)
min(p.rankSMDall)
max(p.rankSMDall)

sum(p.rankSMDpub>0.05)
sum(p.rankSMDpub<0.05)
median(p.rankSMDpub)
min(p.rankSMDpub)
max(p.rankSMDpub)

sum(p.rankRMDallfp<0.05)
sum(p.rankRMDallfp>0.05)
median(p.rankRMDallfp)
min(p.rankRMDallfp)
max(p.rankRMDallfp)

sum(p.rankRMDpubfp>0.05)
sum(p.rankRMDpubfp<0.05)
median(p.rankRMDpubfp)
min(p.rankRMDpubfp)
max(p.rankRMDpubfp)

sum(p.rankSMDallfp<0.05)
sum(p.rankSMDallfp>0.05)
median(p.rankSMDallfp)
min(p.rankSMDallfp)
max(p.rankSMDallfp)


sum(p.rankSMDpubfp>0.05)
sum(p.rankSMDpubfp<0.05)
median(p.rankSMDpubfp)
min(p.rankSMDpubfp)
max(p.rankSMDpubfp)

sum(p.rankNMDall<0.05)
sum(p.rankNMDall>0.05)
median(p.rankNMDall)
min(p.rankNMDall)
max(p.rankNMDall)

sum(p.rankNMDpub>0.05)
sum(p.rankNMDpub<0.05)
median(p.rankNMDpub)
min(p.rankNMDpub)
max(p.rankNMDpub)

sum(p.rankNMDallfp<0.05)
sum(p.rankNMDallfp>0.05)
median(p.rankNMDallfp)
min(p.rankNMDallfp)
max(p.rankNMDallfp)

sum(p.rankNMDpubfp>0.05)
sum(p.rankNMDpubfp<0.05)
median(p.rankNMDpubfp)
min(p.rankNMDpubfp)
max(p.rankNMDpubfp)
```

```
#calculate meta-analysis
metaRMDall<-metagen(ES.rmd,rmd.se,data=alldata)
metaRMDpubb<-metagen(ES.rmd,rmd.se, data=pubbiasdata)
metaRMDallfp<-metagen(ES.rmd,n.fp, data=alldata)
metaRMDpubbfp<-metagen(ES.rmd,n.fp, data=pubbiasdata)

metaSMD2all<-metagen(es.d2, d.se2, data=alldata)
metaSMD2pubb<-metagen(es.d2, d.se2, data=pubbiasdata)
metaSMD2allfp<-metagen(es.d2, n.fp, data=alldata)
metaSMD2pubbfp<-metagen(es.d2, n.fp, data=pubbiasdata)

metaNMDall<-metagen(es.nmd, nmd.se, data=alldata)
metaNMDpubb<-metagen(es.nmd, nmd.se, data=pubbiasdata)
metaNMDallfp<-metagen(es.nmd, n.fp, data=alldata)
metaNMDpubbfp<-metagen(es.nmd, n.fp, data=pubbiasdata)

metabias(metaRMDall)
metabias(metaRMDpubb)
metabias(metaRMDallfp)
metabias(metaRMDpubbfp)

metabias(metaSMD2all)
metabias(metaSMD2pubb)
metabias(metaSMD2allfp)
metabias(metaSMD2pubbfp)

funnel(metaRMDall)
funnel(metaRMDpubb)
funnel(metaSMD2all)
funnel(metaSMD2pubb)
funnel(metaSMD2allfp)
funnel(metaSMD2pubbfp)
funnel(metaRMDallfp)
funnel(metaRMDpubbfp)
funnel(metaNMDall)
funnel(metaNMDpubb)
funnel(metaNMDallfp)
funnel(metaNMDpubbfp)

TF<-trimfill(metaRMDall)
trimfill(metaRMDpubb)
trimfill(metaSMD2all)->TF2
trimfill(metaSMD2pubbfp)->TF3
str(TF)
TF3$k0
TF$k0

funnel (trimfill(metaSMD2all))
##RMD's##
pdf("RMDall10_300smallSE.pdf",height=6,width=6)
funnel(metaRMDall, xlab = "Effect size", xlim = c(-7,27),ylab = "Standard Error
 (SE)",bg=1, main = "Simulation (n=12-30)  RMD")
dev.off()

pdf("PublbiasnewRMD10_300.pdf",height=6,width=6)
funnel(metaRMDpubb, xlab = "Effect size", xlim = c(-5,35), ylab = "Standard Error
 (SE)",bg=1, main = "Simulation (n=12-30) with publication bias RMD")
dev.off()

pdf("RMDall10_300smallCORR.pdf",height=6,width=6)
funnel(metaRMDallfp, xlab = "Effect size", xlim = c(-7,27),ylab =
 expression(paste("1/",sqrt(n))),bg=1, main = "Simulation (n=12-30) RMD corrected",
 level=0.00000000000000000001)
dev.off()
```

```
pdf("PublbiasnewRMD10_300CORR.pdf",height=6,width=6)
funnel(metaRMDpubbfp, xlab = "Effect size", xlim = c(-5,30), ylab =
 expression(paste("1/",sqrt(n))),bg=1, main = "Simulation (n=12-30) with publication
 bias RMD", level=0.000000000000000000001)
dev.off()
##SMD's##
pdf("SMDall10_300lsmallSE.pdf",height=6,width=6)
funnel(metaSMD2all, xlab = "Effect size", xlim = c(-0.7,3.4),ylab = "Standard Error
 (SE)",bg=1, main = "Simulation (n=12-30)  SMD")
dev.off()

pdf("PublbiasnewSMD10_300SE.pdf",height=6,width=6)
funnel(metaSMD2pubb, xlab = "Effect size", xlim = c(-0.5,3.5), ylab = "Standard Error
 (SE)",bg=1, main = "Simulation (n=12-30) with publication bias SMD")
dev.off()

pdf("SMDall10_300smallCORR.pdf",height=6,width=6)
funnel(metaSMD2allfp, xlab = "Effect size", level=0.000000000000000000001, xlim = c(-
 0.7,3.4), ylab = expression(paste("1/",sqrt(n))),bg=1, main =
 expression(paste("Simulation (n=12-30) SMD corrected")))
dev.off()

pdf("PublbiasnewSMD10_300CORR.pdf",height=6,width=6)
funnel(metaSMD2pubbfp, xlab = "Effect size", level=0.000000000000000000001, xlim =
 c(-0.5,3.5), ylab = expression(paste("1/",sqrt(n))),bg=1, main =
 expression(paste("Simulation (n=12-30) with publication bias SMD")))
dev.off()
##NMD's##

pdf("NMDall10_300smallSE.pdf",height=6,width=6)
funnel(metaNMDall, xlab = "Effect size", xlim = c(-20,60),ylab = "Standard Error
 (SE)",bg=1, main = "Simulation (n=12-30)  NMD")
dev.off()

pdf("NMDall10_300smallCORR.pdf",height=6,width=6)
funnel(metaNMDallfp, xlab = "Effect size", xlim = c(-20,60),ylab =
 expression(paste("1/",sqrt(n))),bg=1, main = "Simulation (n=12-30) NMD corrected",
 level=0.000000000000000000001)
dev.off()

pdf("PublbiasnewNMD10_300SE.pdf",height=6,width=6)
funnel(metaNMDpubb, xlab = "Effect size", xlim = c(-10,65), ylab = "Standard Error
 (SE)",bg=1, main = "Simulation (n=12-30) with publication bias NMD")
dev.off()

pdf("PublbiasNMDnew10_300CORR.pdf",height=6,width=6)
funnel(metaNMDpubbfp, xlab = "Effect size", xlim = c(-10,65), ylab = "Standard Error
 (SE)",bg=1, main = "Simulation (n=12-30) with publication bias
 NMD",level=0.000000000000000000001)
dev.off()

metabias(metaSMD2all)
metabias(metaSMD2pubb)
metabias(metaSMD2allfp)
metabias(metaSMD2pubbfp)

metabias(metaSMD2)
metaSMD2
trimfill(metaSMD2, ma.fixed=FALSE)

metabias(metaRMD)
metaRMD
trimfill(metaRMD, ma.fixed=FALSE)
```

```
##metabias(metaSMD)  #cohen's d
##trimfill(metaSMD, ma.fixed=FALSE)

metabias(metaSMDfp)
metaSMDfp
trimfill(metaSMDfp, ma.fixed=FALSE)

metabias(metaNMD)
metaNMD
trimfill(metaNMD,ma.fixed=FALSE)

funnel(metaRMD, studlab=TRUE, lty.fixed = 3, lty.random = 5, xlim = c(-12,12), xlab =
 "Effect size", ylim= c(6,0), ylab = "Standard Error (SE)", cex = .75, col = 1, bg =
 1, contour=c(0.1, 0.01), col.contour=c("grey", "white"), main = "RMD (Δμ=0) 30
 studies")
funnel(metaSMD, studlab=TRUE, lty.fixed = 3, lty.random = 5, xlim = c(-1.2,1.2), xlab
 = "Effect size", ylab = "Standard Error (SE)", cex = .75, col = 1, bg = 1,
 contour=c(0.1, 0.01), col.contour=c("grey", "white"), main = "SMD (Δμ=10) 30
 studies")

funnel(metaSMD, studlab=TRUE, lty.fixed = 3, lty.random = 5, xlim = c(-1.2,1.2), xlab
 = "Effect size", ylab = "Standard Error (SE)", cex = .75, col = 1, bg = 1,
 contour=c(0.1, 0.01), col.contour=c("grey", "white"), main = "SMD (Δμ=10) 30
 studies")

funnel(metaSMDfp, lty.fixed = 3, lty.random = 5, xlim = c(-1,4.2), xlab = "Effect
 size", ylab = "1 / n participants", cex = .75, col = 1, bg = 1, contour=c(0.1,
 0.01), col.contour=c("grey", "white"), main = "SMD corrected (Δμ=10) 3000 studies")
funnel(metaRMD, lty.fixed = 3, lty.random = 5, xlim = c(-1,50), xlab = "Effect size",
 ylab = "1 / n participants", cex = .75, col = 1, bg = 1, contour=c(0.1, 0.01),
 col.contour=c("grey", "white"), main = "SMD corrected (Δμ=10) 3000 studies")

ggplot (as.data.frame(ES.rmd), aes(ES.rmd)) +geom_histogram (colour="black",
 fill="grey", binwidth=0.5)
ggplot (as.data.frame(es.d), aes(es.d)) +geom_histogram (colour="black", fill="grey",
 binwidth=0.5)

shapiro.test(es.d)
shapiro.test(ES.rmd)
ks.test(es.d,pnorm)
ks.test(ES.rmd,pnorm)
metaSMD2 #show meta-analysis
metaRMD
#compare against a t-test
t.test(x, y, alternative = "two.sided", paired = FALSE, var.equal = TRUE, conf.level
 = 0.95) #t-test to compare against meta-analysis
forest(metaRMD, leftcols=c("studlab"), studlab=TRUE, xlab="Raw Mean Difference",
 col.square="#E69F00", col.square.lines="black", col.i="black", fontsize=6)
forest(metaSMD2, leftcols=c("studlab"), studlab=TRUE, xlab="Standardized Mean
 Difference", col.square="#E69F00", col.square.lines="black", col.i="black",
 fontsize=6)

##PUBLICATION BIAS / FUNNELPLOTS##

pdf("RMD_10 3000.pdf", height=6, width=6)
funnel(metaRMD, xlab = "Effect size", ylab = "Standard Error (SE)", bg = 1, main =
 expression(paste("RMD (", Delta,mu, "=10) 3000 studies")))
dev.off()

##OLD DATASETS WITH SMDs REVISED
##EXTRA CAMARADES/SYRCLE DATASETS##
```

```
setwd("/Users/peterpaulzw/Google Drive/Promotie/Projects/meta funnel plot
 asymmetry/Datasets")

##
read.xlsx("peterpaulSMDRMD.xlsx","EF_small")->metapp

metappRMD <-metagen(DifferenceEF,RawMeanDiff_SE,data=metapp)
trimfill(metappRMD)
funnel(metappRMD,xlim = c(-1,8), xlab = "SMD", ylab = "Standard Error",bg=1, main =
 "Dataset 2(Zwetsloot)")

metabias(metatest2SMD)
trimfill(metatest2SMD, left=TRUE, ma.fixed=FALSE)->trimfillSMDtest2
pdf("GroeninkSMD2.pdf",height=6,width=6)
funnel(trimfillSMDtest2,xlim = c(-1,8), xlab = "SMD", ylab = "Standard Error",bg=1,
 main = "Dataset 3(Groenink)")
dev.off()
trimfillSMDtest2

metabias(metatest2SMDad)
trimfill(metatest2SMDad, left=TRUE, ma.fixed=FALSE)->trimfillSMDadtest2
trimfillSMDadtest2
pdf("GroeninkSMD2ad.pdf",height=6,width=6)
funnel(trimfillSMDadtest2, xlim=c(-1,8), xlab = "SMD", ylab =
 expression(paste("1/",sqrt(n))),bg=1, main = "Dataset
 3(Groenink)",level=0.0000000000000000001)
dev.off()

##
read.xlsx("published SMD data for re-analysis.xlsx","SR Kleikers2015 infarct size")-
 >metaother4

metatest4SMD <-metagen(SMD,SMD.SE,data=metaother4)
metatest4SMDad <-metagen(SMD,precision2,data=metaother4)

metabias(metatest4SMD)
trimfill(metatest4SMD, left=FALSE, ma.fixed=FALSE)->trimfillSMDtest4
trimfillSMDtest4
pdf("KleikersSMD.pdf",height=6,width=6)
funnel(trimfillSMDtest4, xlim=c(-6,6), xlab = "SMD", ylab = "Standard Error",bg=1,
 main = "Dataset 4(Kleikers)",level=0.0000000000000000001)
dev.off()

metabias(metatest4SMDad)
trimfill(metatest4SMDad, left=FALSE, ma.fixed=FALSE)->trimfillSMDadtest4
trimfillSMDadtest4
pdf("KLeikersSMDad.pdf",height=6,width=6)
funnel(trimfillSMDadtest4, xlim=c(-6,6), xlab = "SMD", ylab =
 expression(paste("1/",sqrt(n))),bg=1, main = "Dataset 4(Kleikers)",
 level=0.0000000000000000001)
dev.off()

##
read.xlsx("published SMD data for re-analysis.xlsx","SR Wever 2012 creat")-
 >metaother1
metatestSMD <-metagen(SMD,SMD.SE,data=metaother1)
metatestSMDad <-metagen(SMD,precision2,data=metaother1)

metabias(metatestSMD)
trimfill(metatestSMD, left=TRUE, ma.fixed=FALSE)->trimfillSMDtest
trimfillSMDtest
pdf("kimSMD2.pdf",height=6,width=6)
funnel(trimfillSMDtest, xlim=c(-12,16), xlab = "SMD", ylab = "Standard Error",bg=1,
 main = "Dataset 5(Wever)")
```

```
dev.off()


metabias(metatestSMDad)
trimfill(metatestSMDad, left=TRUE, ma.fixed=FALSE)->trimfillSMDadtest
trimfillSMDadtest
pdf("kimSMD2ad.pdf",height=6,width=6)
funnel(trimfillSMDadtest, xlim=c(-12,16), xlab = "SMD", ylab =
 expression(paste("1/",sqrt(n))),bg=1, main = "Dataset
 5(Wever)",level=0.000000000000000000001)
dev.off()

##
read.xlsx("published SMD data for re-analysis.xlsx","SR Yan2015 bone formation")-
 >metaother3

metatest3SMD <-metagen(SMD,SMD.SE,data=metaother3)
metatest3SMDad <-metagen(SMD,precision2,data=metaother3)

metabias(metatest3SMD)
trimfill(metatest3SMD, left=FALSE, ma.fixed=FALSE)->trimfillSMDtest3
pdf("YanSMD.pdf",height=6,width=6)
funnel(trimfillSMDtest3, xlim=c(-21,30), xlab = "SMD", ylab = "Standard Error
 (SE)",bg=1, main = "Dataset 6(Yan)")
dev.off()
trimfillSMDtest3

metabias(metatest3SMDad)
trimfill(metatest3SMDad, left=FALSE,ma.fixed=FALSE)->trimfillSMDadtest3
pdf("YanSMDad.pdf",height=6,width=6)
funnel(trimfillSMDadtest3, xlim=c(-21,30), xlab = "SMD", ylab =
 expression(paste("1/",sqrt(n))),bg=1, main = "Dataset 6(Yan)",
 level=0.000000000000000000001)
dev.off()

trimfillSMDadtest3

##
read.xlsx("Kieren_DataforKim.xlsx","Raw data")->metaother5
metatest5SMD <-metagen(SMD,SMD.SE,data=metaother5)
metatest5SMDad <-metagen(SMD,precision2,data=metaother5)

metabias(metatest5SMD)
trimfill(metatest5SMD, left=TRUE, ma.fixed=FALSE,method="HS")->trimfillSMDtest5
trimfillSMDtest5
pdf("EganSMD.pdf",height=6,width=6)
funnel(trimfillSMDtest5, xlim=c(-35,35), xlab = "SMD", ylab = "Standard Error",bg=1,
 main = "Dataset 7(Egan)")
dev.off()

metabias(metatest5SMDad)
trimfill(metatest5SMDad, left=FALSE, ma.fixed=FALSE)->trimfillSMDadtest5
pdf("EganSMDad_extra.pdf",height=6,width=6)
funnel(trimfillSMDadtest5, xlim=c(-35,35), xlab = "SMD", ylab =
 expression(paste("1/",sqrt(n))),bg=1, main = "Dataset 7(Egan)",
 level=0.000000000000000000001)
dev.off()

trimfillSMDadtest5
```
