# Supplementary material for: Standardized mean differences cause funnel plot distortion in publication bias assessments
Source: eLife. 2017 Sep 8;6:e24260. doi: 10.7554/eLife.24260 (PMC5621838; doi:10.7554/eLife.24260)
Supplement: Transparent reporting form [file elife-24260-transrepform.pdf]

eLIFE
1st Floor
24 Hills Road
Cambridge CB2 1JP, UK
P 01223 855340
W elifesciences.org
T @elife

eLife's transparent reporting form

We encourage authors to provide detailed information *within their submission* to facilitate the interpretation and replication of experiments. If you have any questions, please contact us: editorial@elifesciences.org.

Sample-size estimation

- You should state whether an appropriate sample size was computed when the study was being designed
- You should state the statistical method of sample size computation and any required assumptions
- If no explicit power analysis was used, you should describe how you decided what sample (replicate) size (number) to use

Please outline where this information can be found within the submission (e.g., page numbers or figure legends), or explain why this information doesn't apply to your submission:

Rationale for the number of studies in the simulated meta-analyses is reported in materials and methods, p24, under "data simulations".

Replicates

- You should report how often each experiment was performed
- You should include a definition of biological versus technical replication
- The data obtained should be provided and sufficient information should be provided to indicate the number of independent biological and/or technical replicates
- If you encountered any outliers, you should describe how these were handled
- Criteria for exclusion/inclusion of data should be clearly stated
- High-throughput sequence data should be uploaded before submission, with a private link for reviewers provided (these are available from both GEO and ArrayExpress)

Please outline where this information can be found within the submission (e.g., page numbers or figure legends), or explain why this information doesn't apply to your submission:

Replication of simulations is reported in materials and methods, p23-24, under "data simulations".
Biological or technical replications do not apply to these meta-analysis data.
No outlier analysis or removal of outliers was performed, all datasets were analyzed and reported completely.
No inclusion or exclusion criteria were applied to the data.
No high-throughput sequence data are described in this manuscript.

eLife Sciences Publications, Ltd is a limited liability non-profit non-stock corporation incorporated in the State of Delaware, USA, with company number 5030732, and is registered in the UK with company number FC030576 and branch number BR015634 at the address 1st Floor, 24 Hills Road, Cambridge CB2 1JP | August 2014

Statistical reporting

- Statistical analysis methods should be described and justified
- Raw data should be presented in figures whenever informative to do so (typically when N per group is less than 10)
- For each experiment, you should identify the statistical tests used, exact values of N, definitions of center, methods of multiple test correction, and dispersion and precision measures (e.g., mean, median, SD, SEM, confidence intervals; and, for the major substantive results, a measure of effect size (e.g., Pearson's r, Cohen's d)
- Report exact p-values wherever possible alongside the summary statistics and 95% confidence intervals. These should be reported for all key questions and not only when the p-value is less than 0.05.

Please outline where this information can be found within the submission (e.g., page numbers or figure legends), or explain why this information doesn't apply to your submission:

Statistical analyses are described in materials and methods section, p23-25
Individual (raw) data points for example funnel plots are presented in all figures.
Exact p-values are reported whenever appropriate (results 2.1, table 3, table 4)
Differences between simulation scenarios (e.g. RMD, SMD, NMD) were not tested statistically.

(For large datasets, or papers with a very large number of statistical tests, you may upload a single table file with tests, Ns, etc., with reference to page numbers in the manuscript.)

Additional data files ("source data")

- We encourage you to upload relevant additional data files, such as numerical data that are represented as a graph in a figure, or as a summary table
- Where provided, these should be in the most useful format, and they can be uploaded as "Source data" files linked to a main figure or table
- Include model definition files including the full list of parameters used
- Include code used for data analysis (e.g., R, MatLab)
- Avoid stating that data files are "available upon request"

Please indicate the figures or tables for which source data files have been provided:

As reported in the materials and methods section, data simulations were replicated 1000 times. We show example funnel plots for these simulations, as well as aggregated data on their outcomes. The R script with which all of our simulations can be repeated is reported in the supplementary material.
Data of the meta-analyses used for re-analyses are currently available through the original review authors.
